# Mechanical Predictors of Discomfort during Load Carriage

**DOI:** 10.1371/journal.pone.0142004

**Published:** 2015-11-03

**Authors:** Patrick D. Wettenschwiler, Silvio Lorenzetti, Rolf Stämpfli, René M. Rossi, Stephen J. Ferguson, Simon Annaheim

**Affiliations:** 1 Empa, Swiss Federal Laboratories for Materials Science and Technology, St. Gallen, Switzerland; 2 Institute for Biomechanics, ETH Zurich, Zurich, Switzerland; Université de Technologie de Compiègne, FRANCE

## Abstract

Discomfort during load carriage is a major issue for activities using backpacks (e.g. infantry maneuvers, children carrying school supplies, or outdoor sports). It is currently unclear which mechanical parameters are responsible for subjectively perceived discomfort. The aim of this study was to identify objectively measured mechanical predictors of discomfort during load carriage. We compared twelve different configurations of a typical load carriage system, a commercially available backpack with a hip belt. The pressure distribution under the hip belt and the shoulder strap, as well as the tensile force in the strap and the relative motion of the backpack were measured. Multiple linear regression analyses were conducted to investigate possible predictors of discomfort. The results demonstrate that static peak pressure, or alternatively, static strap force is a significant (p<0.001) predictor of discomfort during load carriage in the shoulder and hip region, accounting for 85% or more of the variation in discomfort. As an additional finding, we discovered that the regression coefficients of these predictors are significantly smaller for the hip than for the shoulder region. As static peak pressure is measured directly on the body, it is less dependent on the type of load carriage system than static strap force. Therefore, static peak pressure is well suited as a generally applicable, objective mechanical parameter for the optimization of load carriage system design. Alternatively, when limited to load carriage systems of the type backpack with hip belt, static strap force is the most valuable predictor of discomfort. The regionally differing regression coefficients of both predictors imply that the hip region is significantly more tolerant than the shoulder region. In order to minimize discomfort, users should be encouraged to shift load from the shoulders to the hip region wherever possible, at the same time likely decreasing the risk of low back pain or injury.

## Introduction

Discomfort during load carriage is a major issue for activities using backpacks (e.g. infantry maneuvers, children carrying school supplies, or outdoor sports). According to Sheir-Neiss et al. [1], 74% of adolescent backpack wearers suffer from neck or back pain, validated by significantly poorer general health, more limited physical functioning, and more bodily pain. As loads have increased during the last decades [2, 3], discomfort during load carriage will become an even more important topic. With increasing loads, discomfort is more likely to be accompanied by injuries like the impairment of the brachial plexus [4–8] or low back pain or injury [9–11]. Any efforts to reduce the occurrence of these medical issues, ranging from discomfort to severe injury, are thus most welcome. A potential improvement could be achieved by optimizing load carriage system design, e.g. system structure or the material properties at the interface between the system and the body. Partial success towards this goal has already been achieved through the use of load carriage systems with a hip belt or a comparable structure, supporting a load shift from the shoulders to the hip [12]. Manufacturers of load carriage systems have an additional motivation to improve their design, as discomfort is known to have considerable influence on user acceptance [13, 14]. However, the variety of currently available load carriage systems suggests that the optimum has not yet been reached. The optimization is hindered by the fact that discomfort is a subjective perception and also depends on the length of time the system is worn [15]. In addition, the personal mood of the subjects and their physical constitution may influence the subjective perception of discomfort [16, 17]. Therefore, objective measurement of discomfort during load carriage is currently very challenging.

There are several definitions of comfort and discomfort available in the literature [18]. According to Richards et al. [19], “Comfort is a continuous dimension of experience–varying from strongly positive (very comfortable) to strongly negative (very uncomfortable).” Discomfort depends on biomechanical factors that are responsible for feelings of pain, soreness, numbness, stiffness and other comparable perceptions [20, 21]. Additionally, thermal aspects may influence discomfort, but they are well understood [22, 23]. In contrast, little is known about the mechanical aspects of discomfort: Local ischemia in the skin and underlying muscular and neural tissue is expected to induce discomfort: Holloway et al. [24] reported blood occlusion to occur at a body surface pressure of 16 kPa. Sangeorzan et al. [25] suggested even lower pressure values between 5.6 kPa and 9.5 kPa to be sufficient for ischemia. The direct compressive force of a backpack’s shoulder strap was shown to induce increased local fatigue in the upper trapezius muscle during an exhausting arm abduction task [26]. Regarding recommended limits for mechanical parameters, Bryant et al. [27] investigated a static scenario and proposed a maximal lumbar force of 135 N and a maximal shoulder force of 145 N for load carriage. The same study reported that an average pressure of 20 kPa in the shoulder region resulted in discomfort for 90% of the soldiers examined. In light of these scarce findings in the literature, it is not yet fully understood which mechanical parameters are directly responsible for subjectively perceived discomfort. There are many open questions, e.g. which parameters have the largest influence on discomfort? Furthermore, is it required to differentiate between static and dynamic values of mechanical parameters like pressures and forces? It is evident that gaining a comprehensive knowledge of the mechanical predictors is a crucial step towards minimizing discomfort during load carriage. Therefore, the aim of this study was to identify objectively measured mechanical predictors of discomfort during load carriage, using pressure sensing mats, strap force sensors and a 3D motion tracking system.

## Materials and Methods

Ten male subjects without any history of back pain and with the following anthropometrical characteristics were tested (mean ± standard deviation): age 28.0 ± 3.7 years, weight 73.1 ± 10.4 kg, height 178.7 ± 5.7 cm. All subjects provided written informed consent.

This study was approved by the Ethical Committee of the Canton of St. Gallen and was carried out in accordance with “The Code of Ethics of the World Medical Association” (Declaration of Helsinki, amended October 2013).

### Load Carriage System

The load carriage system applied in this study is the commercially available backpack “Deuter ACT Lite 50+10” (Deuter Sport GmbH, Gersthofen, Germany). According to the manufacturer, it is intended for use in a wide variety of activities, including trekking, alpine tours and travelling. Two modifications were made to the system for this study: Firstly, all metal parts had to be removed due to the use of electromagnetic sensors. Secondly, an efficient change of the payload, while keeping the center of mass constant in all three dimensions, had to be enabled.

#### Modifications

**Fig 1:**
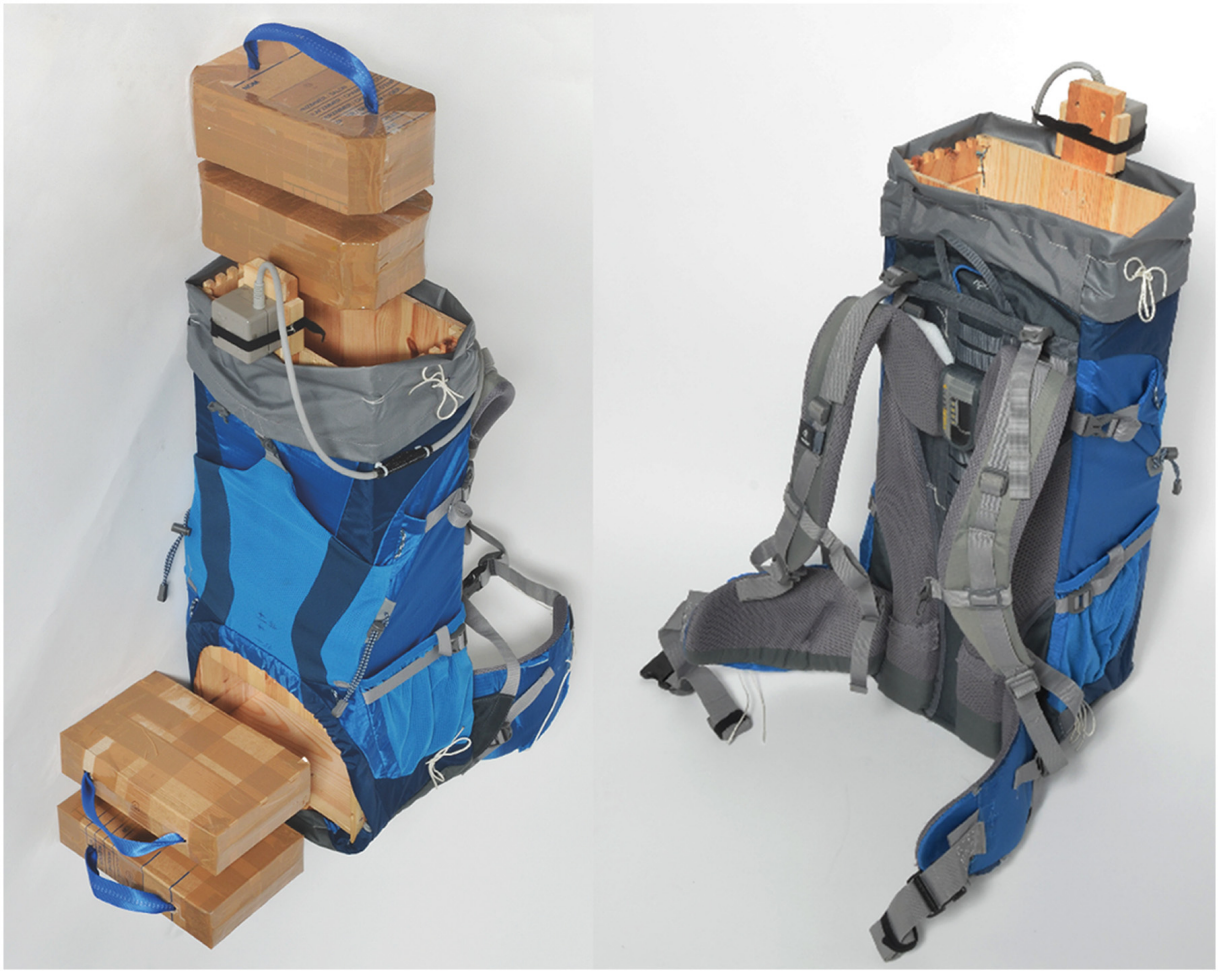
Modified load carriage system. The modular payload is shown on the left.

All modifications respected lateral symmetry of the backpack. We removed the aluminum rods, which built the frame in the back wall of the backpack. A wooden box (65.0 cm height, 27.5 cm length, 15.8 cm depth, and 0.9 cm thickness) was inserted into the backpack and fixed to the back wall of the backpack. The rigid connection between the backpack and the wooden box replaced the function of the aluminum rods, enabling load transfer between the hip belt and the shoulder straps. Two openings in the backpack and the wooden box were created for easy access to a modular payload, one at the bottom and one at the top. The payload was constructed out of cardboard boxes filled with sand and resulted in a steady center of mass, positioned 30.5 cm from the bottom (Fig 1).

#### Configurations

We compared twelve different configurations of one typical load carriage system, thus eliminating potential effects of the design on discomfort. Mackie et al. [28] reported that load weight and hip belt use have the largest effects on interface pressure and strap forces. Therefore, we defined twelve configurations resulting from a combination of three different loads and four different hip belt lengths: The total masses of the load carriage system were 15.0 kg, 20.0 kg, and 25.0 kg. The hip belt lengths were calibrated for each subject, corresponding to 30 N, 60 N, 90 N, and 120 N of tension during initial upright standing with the 20.0 kg load. As the tension in the hip belt is sensitive to breathing and hip joint motion, hip belt length is considered to be more appropriate than hip belt tension to define the load carriage system configurations.

### Experiments

For all measurements, subjects wore running shorts and sneakers, but no shirt. The back length of the load carriage system was adjusted for each subject to position the upper end of the hip belt level with the highest point of the iliac crest. The lengths of the hip belt were marked according to the predefined tension levels. In an acclimatization phase, the subjects walked with the load carriage system on the treadmill at 4.5 km/h, until they felt at ease. One subject could not adjust to comfortable walking at this speed, and instead felt comfortable walking at 4.0 km/h. This subject consequently performed all measurements at 4.0 km/h. As soon as the subjects felt at ease, they were asked to stop and to fill out the discomfort questionnaire according to the currently perceived discomfort. These answers were not evaluated, as they served as a familiarization trial.

To minimize the effect of minor differences in the exact placement of the load carriage system, three iterations were performed to measure the mechanical parameters, with a break of 20 minutes in between. These iterations were identical, except for the order in which the load carriage system configurations were assessed: Firstly, as a warm-up, subjects walked on the treadmill for two minutes with a total load of 15.0 kg. Then one measurement was performed without the load carriage system, to record possible artifacts in the pressure sensors due to bending. The reason for this measurement without load is explained in more detail in the section “Body Surface Pressure” below. Afterwards, all load carriage system configurations were applied in a randomized order. All randomizations for our study were based on true random numbers provided by RANDOM.ORG [29]. For every configuration, a static measurement was followed by a dynamic measurement, each of them lasting for six seconds at a sampling rate of 120 Hz. During the static measurement, subjects stood upright, facing straight ahead. During the dynamic measurement, subjects walked on the level treadmill. The dynamic measurement was triggered by the investigator simultaneously with the right heel strike of the subjects, as soon as they were walking regularly at the given speed.

After completing the measurements of the mechanical parameters, the subjects were asked to report their discomfort for each configuration in randomized order. For this discomfort assessment, the subjects walked on the treadmill for one minute with each configuration, before stopping and filling out the discomfort questionnaire regarding the currently perceived discomfort with the corresponding configuration. By assessing the discomfort at the very end of the experiments and choosing a small duration for the last treadmill walk with each configuration, we prevented substantial differences in total prior exposition time between the configurations.

### Measured Parameters

#### Body surface pressure

**Fig 2:**
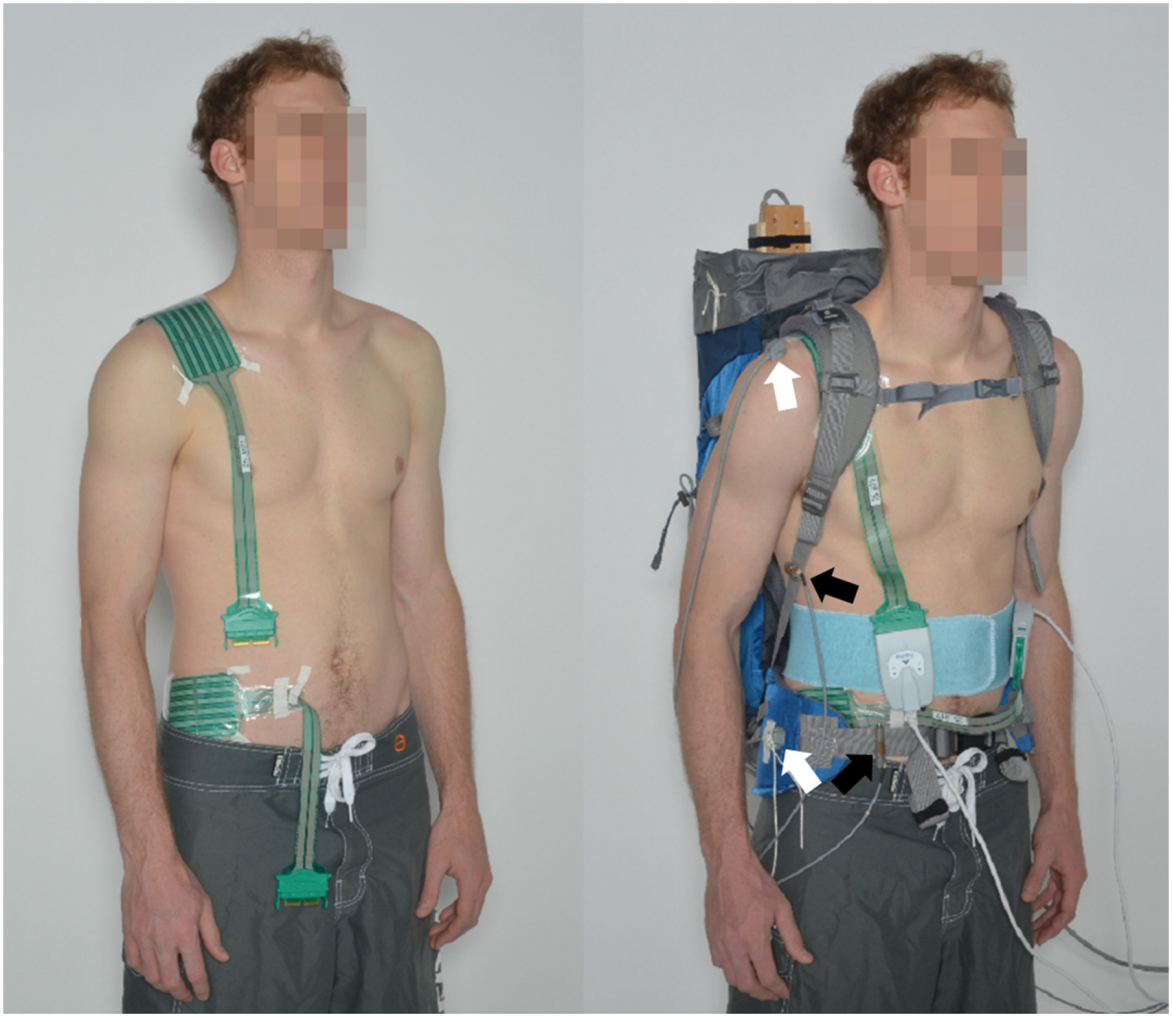
Subject with pressure sensors (left) and load carriage system (right). On the right side, black arrows point to the locations of the strap force sensors on the shoulder strap and on the hip belt and white arrows point to the locations of the Polhemus sensors on the shoulder and on the hip.

Body surface pressure was recorded using Tekscan type 9811E pressure sensitive foils (Tekscan, South Boston, MA, USA) with a pressure range up to 172 kPa, according to the manufacturer’s specifications. The sensitive foil comprises 6 x 16 sensor cells, covering an area of 7.6 cm x 20.3 cm. Prior to use, the sensors were conditioned, equilibrated, and calibrated according to the manufacturer’s recommendations. Equilibration was performed at 20 kPa, while two-point calibration was performed at 20 kPa and 50 kPa. The sensors were placed on the right shoulder and right hip region of the subjects, as shown in Fig 2. The individual in Fig 2 has given written informed consent (as outlined in PLOS consent form) to publish these pictures. For a precise placement of the pressure sensors according to anatomical landmarks and for easier handling, the subjects wore no shirt. The sensitivity of Tekscan sensors to humidity and temperature has been reported in a previous study [30]. To protect the sensors from humidity, the sensors were welded into 0.05 mm thick polyethylene protective covers. As the clothing layers have no influence on the pressure [31], thin polyethylene layers are not expected to have an influence on the pressure. To minimize a possible change in temperature during the measurements, the sensors were worn during the two-minute warm-up walk. Newly calibrated sensors were used for every measurement iteration step.

The recorded pressure distribution data was processed in MATLAB (R2012b, The MathWorks, Natick, MA, USA) to determine the average pressure and the peak pressure of each measurement. With a measurement duration of six seconds and a sampling rate of 120 Hz, every measurement consisted of 720 frames. An offset correction was performed first to account for possible artifacts due to bending of the sensors: Using the measurements without load carriage system, a base average value was calculated over all frames for each cell of the sensors. The base values were subtracted from all corresponding measurements with the load carriage system. After this offset correction, the average pressure and the peak pressure were calculated for static and dynamic measurements in the shoulder and the hip region. The average pressure value was calculated by first taking the mean of all non-zero cells in each measured frame. With these mean values of each frame, it was further possible to calculate the mean value over time to reach the average pressure of each measurement. Similarly, the peak pressure was calculated by first taking the maximum value in each frame. With these maximum values for each frame, it was further possible to extract the maximum across time to reach the peak pressure of each measurement. For each subject, the final average and peak pressures were calculated by taking the mean of all three iterations.

#### Strap Force

The forces were measured in the right shoulder strap and in the hip belt using force sensors based on strain gauges (Fig 2). To determine the strap forces for each measurement, the mean value over time was calculated. For each subject, the strap forces were calculated by taking the mean of all three iterations.

#### Relative Motion

The 3D motion tracking system Polhemus Liberty (Polhemus, Colchester, VT, USA) was used to investigate the relative motion between the bulk of the load carriage system and the body. The electromagnetic field source of the tracking system was mounted at the posterior top of the load carriage system (Fig 1). Using double-sided adhesive tape, sensors were mounted on the acromion of the right shoulder and on the hip fin of the load carriage system at the height of the underlying anterior superior iliac spine (Fig 2). For each measurement, the cumulative change of distance between sensor and source in all three axes, divided by the measurement duration, was calculated for the relative motion value. For each subject, the final relative motion was calculated by taking the mean of all three iteration steps.

#### Discomfort

The focus of this study lies on the mechanical aspects of discomfort, therefore thermal aspects of discomfort were minimized as much as possible. The lab environment (air conditioning) was adjusted to the subjects’ individual preferences and the study design featured only moderate activity.

For the subjective discomfort scores in our study, the subjects were asked to rate the currently perceived discomfort on a visual analogue scale, ranging from “no discomfort”, corresponding to a value of 0, to “maximal discomfort”, corresponding to a value of 10. For every load carriage system configuration, one score for the shoulder region, one for the hip region, and one for the overall discomfort was recorded.

### Statistical Analysis

For each parameter, the mean of all subjects was calculated for every load carriage system configuration in order to exclude the effect of the intra-subject variation in the perception of discomfort. Using these mean values of all subjects, several multiple linear regression analyses were conducted using IBM SPSS (22.0, IBM Corp., Armonk, NY, USA) to identify the mechanical predictors of discomfort (Table 1). In a first approach, only the pressure parameters (average pressure and peak pressure) were entered as independent variables. When using these parameters as possible predictors, the type and design of the load carriage system is not relevant. Therefore the results of these regressions are also valid for other types of load carriage systems. In a second round, all parameters were entered as independent variables. Consequently, these results are only valid for load carriage systems of the type backpack with hip belt. However, rather than conducting a regression analysis with eleven degrees of freedom and eight possible predictors, separate regressions were conducted for the static and dynamic parameters in the second round. All these regressions were calculated for the shoulder and hip region separately.

**Table 1:** List of independent variables included in multiple linear regressions.

		Regression with pressure parameters	Regression with all static parameters	Regression with all dynamic parameters
**Average pressure [kPa]**	**static**	x	x	
	**dynamic**	x		x
**Peak pressure [kPa]**	**static**	x	x	
	**dynamic**	x		x
**Strap force [kPa]**	**static**		x	
	**dynamic**			x
**Relative motion [kPa]**	**static**		x	
	**dynamic**			x

The dependent variable was regional discomfort.

Where the regression results revealed the same single parameter as significant predictor for discomfort in both regions, a direct comparison of both regions is warranted. Hence, the linear relationship of these parameters between the shoulder and the hip region at equal discomfort was calculated from the corresponding regression equations.

Finally, one multiple linear regression was conducted with overall discomfort as dependent variable and with shoulder discomfort and hip discomfort as independent variables. All regression analyses in this study were conducted using backwards elimination method, applying Bonferroni correction for multiple testing. The significance level was defined at p < 0.05.

## Results

The absolute values of the discomfort and the objectively measured mechanical parameters are not the primary interest in this study. Nevertheless, they are provided as a mean of all subjects for all configurations as supporting information (S1–S3 Tables).

**Fig 3:**
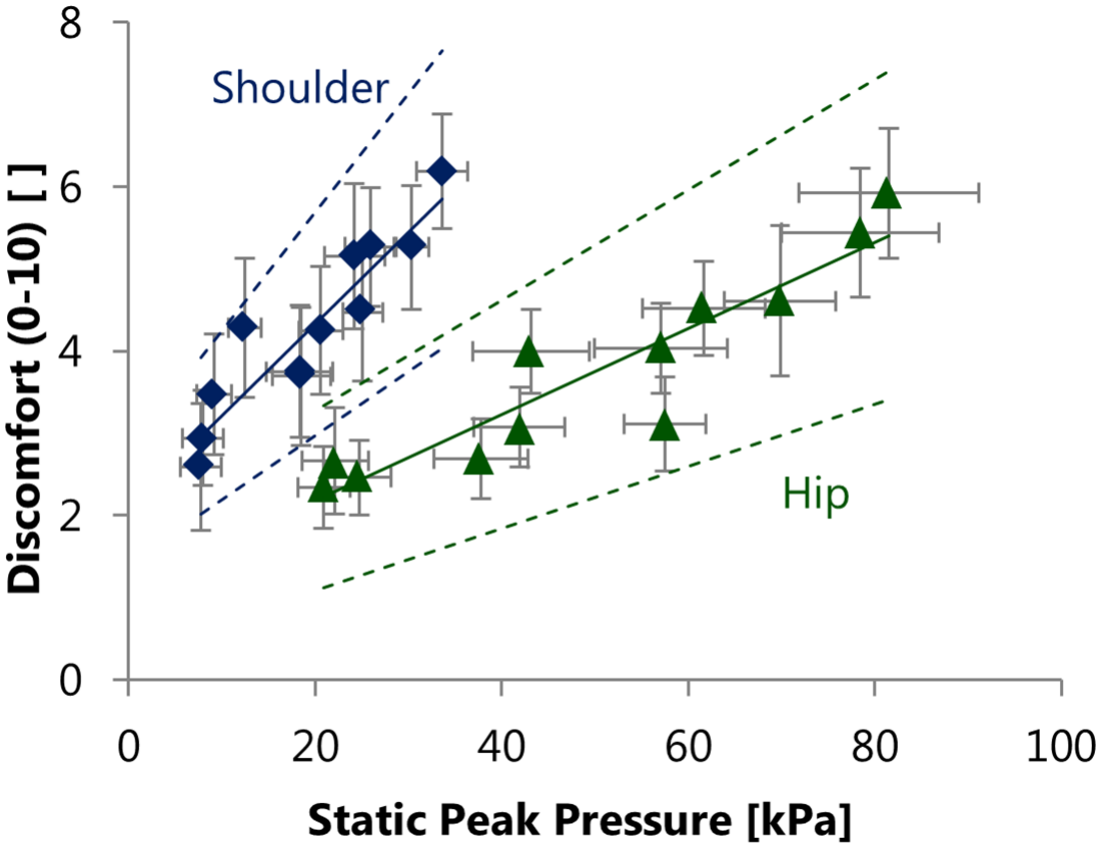
Results of the regression analyses using average and peak pressure (static and dynamic) as independent variables. Multiple linear regression analyses revealed static peak pressure as significant (p<0.001) predictor of discomfort in the shoulder and hip region. The non-significant predictors were removed from the model during the backwards elimination. Data points show the subject’s mean ± standard error of measurement for each configuration, dotted lines show 95% prediction intervals. Regression equations: y = 0.111x + 2.102, R^2^ = 0.85 (shoulder); y = 0.052x + 1.127, R^2^ = 0.86 (hip).

The results of the multiple linear regression analysis in both investigated regions are shown in Fig 3, with discomfort as dependent variable and the pressure parameters as independent variables. The regression coefficients of static peak pressure exhibit the following 95% confidence intervals: 0.038–0.067 for the hip and 0.079–0.144 for the shoulder region.

The results of the multiple linear regression analyses with all mechanical parameters as independent variables are presented separately for the static and dynamic parameters in Table 2.

**Table 2:** Results of the regression analyses using average pressure, peak pressure, strap force, and relative motion as independent variables.

Condition	Region	R^2^	Model	Coefficients	95% confidence interval of coefficients		Standardized coeff.
					lower	upper	
**Static**	**Shoulder**	0.91	Constant	-1.339	-2.590	-0.088	
			Strap force	0.097	0.076	0.118	0.955*
	**Hip**	0.85	Constant	0.884	-0.015	1.782	
			Strap force	0.040	0.029	0.052	0.922*
**Dynamic**	**Shoulder**	0.96	Constant	6.984	2.371	11.596	
			Strap force	0.061	0.043	0.079	0.696*
			Relative motion	-0.836	-1.291	-0.382	-0.375**
	**Hip**	0.94	Constant	-11.275	-19.502	-3.049	
			Strap force	0.041	0.032	0.050	0.909*
			Relative motion	1.049	0.345	1.752	0.286**

The dependent variable was regional discomfort. Only the variables with significant coefficients are listed in the table, the other variables were removed from the model during the backwards elimination.

* p<0.001,

** p<0.01.

For a direct comparison between both regions, the linear relationship between static peak pressure in the shoulder (*p*_*shoulder*_) and in the hip region (*p*_*hip*_) at equal discomfort in both regions is given in Eq (1). The linear relationship between static strap force in the shoulder (*f*_*shoulder*_) and in the hip region (*f*_*hip*_) at equal discomfort in both regions is given in Eq (2).

$$p_{hip}=2.135p_{shoulder}+18.750$$ (1)

$$f_{hip}=2.425f_{shoulder}-55.575$$ (2)

The results of the multiple linear regression analysis with overall discomfort as dependent variable and regional discomfort as independent variables are shown in Table 3.

**Table 3:** Results of the regression analysis using shoulder discomfort and hip discomfort as independent variables.

Model	Coefficients	95% confidence interval of coefficients		Standardized coefficients
		lower	upper	
**Constant**	-0.501	-1.671	0.652	
**Shoulder discomfort**	0.967	0.476	0.865	0.832*
**Hip discomfort**	0.463	0.292	0.634	0.652*

The dependent variable was overall discomfort. R^2^ = 0.90.

*p<0.001.

## Discussion

The aim of this study was to identify objectively measured mechanical predictors of discomfort during load carriage. We compared twelve configurations of a typical load carriage system, evaluating average pressure, peak pressure, strap forces and relative motion between the bulk of the system and the body in the shoulder and hip region for static and dynamic conditions. We conducted the main multiple linear regression analyses twice: once using only the pressure parameters as independent variables and once using all mechanical parameters as independent variables. For both versions, we used regional discomfort as dependent variables.

### Static Peak Pressure as Predictor of Discomfort

Most of the static peak pressure values found in this study are higher than 16 kPa, at which Holloway et al. [24] reported blood occlusion to occur (Fig 3). Consequently, the presence of discomfort in this study is consistent with expectations.

Our results show that static peak pressure, as significant predictor of discomfort, accounts for 85% of discomfort in the shoulder region and for 86% of discomfort in the hip region (Fig 3). These results indicate that mechanical parameters are more powerful predictors of discomfort than literature has previously suggested. In a similar study, Stevenson et al. [32] found average pressure to be the best predictor of discomfort, accounting for 31% of its variation. Compared to the results found in our study, this value reported in literature is lower, which could be due to differences in the design. While the data of Stevenson et al. [32] originated from a comparison of nine different load carriage systems, our study compared twelve different configurations of one load carriage system. Additionally, Stevenson et al. [32] measured the mechanical parameters on a human load carriage simulator, while our measurements were conducted on subjects. Both designs are justified by their respective advantages. A human simulator usually provides better repeatability, whereas human subjects are more realistic. The use of different load carriage systems provides a broader generalization of the findings, whereas by using only one system, as in this study, several otherwise uncontrolled parameters are kept constant. At the same time, we are fully aware that the type of the investigated load carriage system may influence the outcome of a study. However, by using only the pressure parameters for these regressions, we strove to minimize dependence of the measurements on the load carriage system type. The perception of static peak pressure on the body surface does not depend on the type of load carriage system used. Hence, static peak pressure is well suited as generally applicable, objective mechanical parameter for the optimization of load carriage system design. Such an objective mechanical parameter is of great value for the improvement of load carriage system design, so that in the future, users may profit from decreased discomfort during load carriage.

### Strap Force and Relative Motion as Predictors of Discomfort

To investigate whether the mechanical parameters that were measured on the load carriage system could explain additional variance in discomfort, we further conducted multiple linear regression analyses with all mechanical parameters as independent variables.

In static conditions, strap force is a significant (p<0.001) predictor of discomfort in the shoulder and the hip region (Table 2). Therefore, static peak pressure is not the best predictor of discomfort, if static strap force data is considered simultaneously. Static peak pressure does not account for any significant additional variance in discomfort either and is excluded from the models. The explanation for these results lies in the presence of high multicollinearity; among the parameters static average pressure, dynamic average pressure, static peak pressure, dynamic peak pressure, static strap force, and dynamic strap force, the Pearson correlation coefficient was above 0.9 for all comparisons. All regression results contain only one of these parameters. As a consequence, in the absence of static peak pressure or strap force values, other parameters of this group could also be significant predictors of discomfort. Our regression results are not coincidental, as the applied backwards elimination method eliminates the least significant predictor in each step, until all of the remaining predictors meet the predefined significance criterion. One must keep in mind that excluded parameters, like static average pressure, dynamic average pressure, and dynamic peak pressure, are not necessarily invalid as predictors of discomfort. In our sample, they are simply less powerful than static peak pressure or strap force, which we found to be the best predictors of discomfort.

In dynamic conditions, strap force (p<0.001) and relative motion (p<0.01) between the bulk of the load carriage system and the body are significant predictors of discomfort (Table 2). For both regions, relative motion is a less important predictor of discomfort than strap force, as can be seen by the standardized coefficients (Table 2). While more relative motion is associated with more discomfort for the hip region, the opposite was found for the shoulder region. More relative motion in the shoulder region was therefore associated with less discomfort. A possible explanation for this unexpected finding can be found in the results of Sharpe et al.[33]: Compared to the natural walking pattern without load, a backpack reduces the relative phase of the rotation between pelvis and thorax [33]. An increasing relative motion in the shoulder region may therefore be a sign of less restriction of the shoulder girdle through the load carriage system, enabling a more natural motion pattern and resulting in less discomfort. Nevertheless, despite explaining 96% (shoulder) and 94% (hip) of variation in discomfort, our models with dynamic strap force and dynamic relative motion as predictors have to be treated with care. In static conditions, in contrast, the models with strap force as sole significant predictor are conclusive. A big advantage of strap forces is that they are usually much easier to measure than static peak pressures. While these results should not be generalized to any possible type of load carriage system, we consider them to be valid for systems that are comparable to the type used in this study, i.e. backpacks with hip belts. Thus static strap forces can be regarded as valuable predictors of discomfort in both regions when applied to load carriage systems of the type backpack with hip belt.

### Regional Differences in the Perception of Discomfort

Aside from providing objective mechanical parameters to predict discomfort during load carriage, our results also offer some valuable information about optimal adjustments of load carriage systems.

Due to the non-overlapping confidence intervals of the regression coefficients of static peak pressure in the shoulder and the hip region, we can deduce that the hip region is significantly more tolerant than the shoulder region, regarding an increase in static peak pressure. The same is true for static strap force (Table 2). According to the quantitative comparison in Eq (1), static peak pressure in the hip needs to be more than twice the static peak pressure in the shoulder to cause the same amount of discomfort. These results are in line with the findings of Scribano et al. [34], who reported the hip region to be two to three times more tolerant, regarding the absolute values of local skin pressure. More recently, Martin et al. [35] suggested the hip region to be less sensitive than the shoulder region to an increase in pressure. We were able to confirm the finding of Martin et al. (see Eq (1)). The hip region is also less sensitive than the shoulder region to an increase in static strap force (see Eq (2)). In order to minimize discomfort, users should therefore be encouraged to shift load from the shoulders to the hip region wherever possible. This is accompanied by another benefit, because as more load is applied at the hip, less load impacts on the spine. Hence, shifting load to the hip at the same time likely decreases the risk of low back pain or injury.

### Overall Discomfort

As this study focused on discomfort in the shoulder and hip region only, it was important to analyze their association with overall discomfort. Shoulder discomfort and hip discomfort are both significant (p<0.001) predictors of overall discomfort (Table 3). Together, they can explain 90% of the variation in overall discomfort. From these results, we deduce that for this study, focusing on the shoulder and hip region was adequate to draw conclusions also on overall discomfort.

### Limitations

The subjects used in this study were all male and young (28.0 ± 3.7 years). It is not clear to which extent our findings apply also to females and/or older people. Potential factors of variability within the sample of young male subjects include body composition and regional subcutaneous fat distribution. Their influence remains to be investigated in future studies. In addition, the pressure measurements conducted in this study only recorded pressure normal to the body surface. Tangential stress and resulting shear strain in the skin and the underlying tissue is not included in the pressure parameters. However, other parameters, e.g. strap forces, do not differentiate between vertical and tangential pressure and thus shear strain is indirectly considered in our investigations. The most challenging issue was a possible temperature difference of the pressure sensors between calibration and measurement on the skin. To minimize this, sensors were given enough time to adjust to skin temperature and the order of configurations tested was randomized. This enabled an unbiased comparison between configurations using the mean of all subjects. Regarding the load carriage system used in our study, the necessary modifications may have influenced the discomfort perceived by the subjects. Therefore, the discomfort values reported in this study cannot be applied to the commercially available load carriage system in its original state. Finally, instead of letting the subjects walk at a self-selected walking speed, we asked the subjects to walk at 4.5 km/h, as a uniform walking speed naturally occurs in typical fields of load carriage, e.g. trekking/hiking in a group, infantry. One subject could not adjust to comfortable walking at 4.5 km/h. Instead of enforcing an uncomfortable and possibly unnatural walking pattern, we measured this subject at a walking speed of 4.0 km/h. The statistical power of this study is affected by the assumption of linear relationships between discomfort and the predictors, the use of mean values of all subjects, and the confined number of ten subjects.

## Conclusions

In this study, we found that static peak pressure, or alternatively, static strap force, is a significant (p<0.001) predictor of discomfort during load carriage in the shoulder and hip region, accounting for 85% or more of the variation in perceived discomfort. As an additional finding, we discovered that the regression coefficients of these predictors are significantly smaller for the hip than for the shoulder region. For a quantitative comparison, the linear relationship of static peak pressure and static strap force between both regions at equal discomfort was evaluated. This revealed the shoulder region to be more than twice as sensitive as the hip region to an increase in static peak pressure or static strap force. In order to minimize discomfort, users should be encouraged to shift load from the shoulders to the hip region wherever possible, at the same time likely decreasing the risk of low back pain or injury. However, the main outcome of this study is the successful identification of objectively measured mechanical predictors of discomfort, which represent a valuable tool for the optimization of load carriage system design.

## Supporting Information

S1 Table Discomfort scores. For each measured configuration, the mean of all subjects ± the standard error of measurement is shown.
(DOCX)

S2 Table Mechanical parameters in the shoulder region. For each measured configuration, the mean of all subjects ± the standard error of measurement is shown.
(DOCX)

S3 Table Mechanical parameters in the hip region. For each measured configuration, the mean of all subjects ± the standard error of measurement is shown.
(DOCX)
