# Supplementary material for: Mechanical Predictors of Discomfort during Load Carriage
Source: PLoS One. 2015 Nov 3;10(11):e0142004. doi: 10.1371/journal.pone.0142004 (PMC4631336; doi:10.1371/journal.pone.0142004)
Supplement: S1 Table — For each measured configuration, the mean of all subjects ± the standard error of measurement is shown. (DOCX) [file pone.0142004.s001.docx]

**S1 Table. Discomfort scores**.

| **Configuration *** | **Shoulder discomfort** | **Hip discomfort** | **Overall discomfort** |
| --- | --- | --- | --- |
| **1** (15.0 kg, 30 N) | 4.28 ± 0.85 | 2.34 ± 0.50 | 3.40 ± 0.53 |
| **2** (15.0 kg, 60 N) | 3.47 ± 0.73 | 2.69 ± 0.48 | 3.12 ± 0.57 |
| **3** (15.0 kg, 90 N) | 2.94 ± 0.58 | 3.11 ± 0.57 | 2.94 ± 0.39 |
| **4** (15.0 kg, 120 N) | 2.59 ± 0.77 | 4.61 ± 0.92 | 3.35 ± 0.66 |
| **5** (20.0 kg, 30 N) | 5.15 ± 0.88 | 2.66 ± 0.65 | 4.14 ± 0.63 |
| **6** (20.0 kg, 60 N) | 4.25 ± 0.78 | 3.07 ± 0.48 | 3.90 ± 0.59 |
| **7** (20.0 kg, 90 N) | 3.69 ± 0.84 | 4.03 ± 0.55 | 3.72 ± 0.42 |
| **8** (20.0 kg, 120 N) | 3.75 ± 0.80 | 5.44 ± 0.78 | 4.41 ± 0.61 |
| **9** (25.0 kg, 30 N) | 6.19 ± 0.70 | 2.46 ± 0.45 | 5.13 ± 0.50 |
| **10** (25.0 kg, 60 N) | 5.26 ± 0.75 | 3.99 ± 0.51 | 4.49 ± 0.52 |
| **11** (25.0 kg, 90 N) | 5.26 ± 0.72 | 4.52 ± 0.58 | 4.69 ± 0.39 |
| **12** (25.0 kg, 120 N) | 4.47 ± 0.84 | 5.92 ± 0.79 | 5.77 ± 0.53 |

For each measured configuration, the mean of all subjects ± the standard error of measurement is shown.

* The configurations differ in load mass and tension to which the hip belt was adjusted, as shown in brackets.
