# Supplementary material for: Mechanical Predictors of Discomfort during Load Carriage
Source: PLoS One. 2015 Nov 3;10(11):e0142004. doi: 10.1371/journal.pone.0142004 (PMC4631336; doi:10.1371/journal.pone.0142004)
Supplement: S2 Table — For each measured configuration, the mean of all subjects ± the standard error of measurement is shown. (DOCX) [file pone.0142004.s002.docx]

**S2 Table.** **Mechanical parameters in the shoulder region.**

| **Shoulder region** | **Average pressure [kPa]** | | **Peak pressure [kPa]** | | **Strap force [N]** | | **Relative motion [mm/s]** | |
| --- | --- | --- | --- | --- | --- | --- | --- | --- |
| **Configuration *** | static | dynamic | static | dynamic | static | dynamic | static | dynamic |
| **1** (15.0 kg, 30 N) | 6.97 ± 0.98 | 9.28 ± 0.93 | 12.43 ± 1.73 | 24.90 ± 2.32 | 51.93 ± 2.04 | 59.92 ± 2.37 | 0.68 ± 0.10 | 7.83 ± 0.65 |
| **2** (15.0 kg, 60 N) | 4.90 ± 1.12 | 9.89 ± 0.93 | 9.16 ± 1.87 | 23.98 ± 1.86 | 47.96 ± 1.65 | 56.54 ± 2.73 | 0.70 ± 0.09 | 8.39 ± 0.70 |
| **3** (15.0 kg, 90 N) | 4.48 ± 1.17 | 9.41 ± 0.83 | 7.98 ± 2.21 | 22.58 ± 1.90 | 44.99 ± 2.06 | 53.22 ± 2.72 | 0.73 ± 0.11 | 8.47 ± 0.69 |
| **4** (15.0 kg, 120 N) | 4.76 ± 1.32 | 8.60 ± 0.87 | 7.74 ± 2.20 | 20.05 ± 1.90 | 42.29 ± 1.66 | 51.61 ± 2.65 | 0.73 ± 0.08 | 9.14 ± 0.78 |
| **5** (20.0 kg, 30 N) | 10.83 ± 1.27 | 11.69 ± 0.84 | 24.20 ± 3.22 | 34.61 ± 2.53 | 63.23 ± 2.47 | 73.09 ± 2.82 | 0.72 ± 0.08 | 7.63 ± 0.78 |
| **6** (20.0 kg, 60 N) | 9.55 ± 1.53 | 11.50 ± 0.77 | 20.59 ± 2.35 | 37.96 ± 4.61 | 59.26 ± 2.43 | 69.24 ± 3.15 | 0.66 ± 0.08 | 8.02 ± 0.71 |
| **7** (20.0 kg, 90 N) | 8.86 ± 1.48 | 10.98 ± 0.76 | 18.51 ± 3.13 | 30.16 ± 2.36 | 54.13 ± 2.58 | 65.56 ± 3.16 | 0.72 ± 0.08 | 8.31 ± 0.73 |
| **8** (20.0 kg, 120 N) | 8.77 ± 1.62 | 9.82 ± 1.23 | 18.33 ± 3.56 | 28.22 ± 3.78 | 53.21 ± 2.10 | 64.37 ± 2.81 | 0.73 ± 0.09 | 8.86 ± 0.79 |
| **9** (25.0 kg, 30 N) | 12.41 ± 0.97 | 13.15 ± 0.76 | 33.58 ± 2.71 | 44.78 ± 4.06 | 74.21 ± 1.62 | 87.57 ± 2.43 | 0.75 ± 0.14 | 7.56 ± 0.73 |
| **10** (25.0 kg, 60 N) | 13.45 ± 0.98 | 12.73 ± 0.79 | 30.31 ± 1.89 | 43.28 ± 3.48 | 70.48 ± 1.57 | 83.28 ± 3.02 | 0.70 ± 0.08 | 7.89 ± 0.84 |
| **11** (25.0 kg, 90 N) | 9.91 ± 1.06 | 12.65 ± 0.78 | 25.91 ± 2.72 | 39.15 ± 2.63 | 67.77 ± 1.52 | 80.94 ± 2.97 | 0.75 ± 0.10 | 8.21 ± 0.79 |
| **12** (25.0 kg, 120 N) | 12.02 ± 1.20 | 12.32 ± 0.80 | 25.09 ± 2.11 | 37.42 ± 2.99 | 65.69 ± 2.26 | 78.22 ± 2.84 | 0.72 ± 0.10 | 8.38 ± 0.82 |

For each measured configuration, the mean of all subjects ± the standard error of measurement is shown.

* The configurations differ in load mass and tension to which the hip belt was adjusted, as shown in brackets.
