# Supplementary material for: Mechanical Predictors of Discomfort during Load Carriage
Source: PLoS One. 2015 Nov 3;10(11):e0142004. doi: 10.1371/journal.pone.0142004 (PMC4631336; doi:10.1371/journal.pone.0142004)
Supplement: S3 Table — For each measured configuration, the mean of all subjects ± the standard error of measurement is shown. (DOCX) [file pone.0142004.s003.docx]

**S3 Table.** **Mechanical parameters in the hip region.**

| **Hip region** | **Average pressure [kPa]** | | **Peak pressure [kPa]** | | **Strap force [N]** | | **Relative motion [mm/s]** | |
| --- | --- | --- | --- | --- | --- | --- | --- | --- |
| **Configuration *** | static | dynamic | static | dynamic | static | dynamic | static | dynamic |
| **1** (15.0 kg, 30 N) | 11.92 ± 1.78 | 12.68 ± 1.22 | 20.92 ± 2.82 | 28.36 ± 2.40 | 31.85 ± 4.42 | 29.40 ± 3.64 | 3.60 ± 0.44 | 11.40 ± 1.07 |
| **2** (15.0 kg, 60 N) | 14.81 ± 2.31 | 14.64 ± 1.63 | 37.74 ± 5.03 | 45.08 ± 3.72 | 58.87 ± 6.41 | 53.87 ± 5.09 | 3.61 ± 0.47 | 11.49 ± 1.02 |
| **3** (15.0 kg, 90 N) | 16.81 ± 1.94 | 16.43 ± 2.07 | 57.53 ± 4.37 | 61.85 ± 3.82 | 77.79 ± 6.63 | 74.22 ± 4.99 | 3.62 ± 0.47 | 11.14 ± 1.06 |
| **4** (15.0 kg, 120 N) | 18.53 ± 2.07 | 17.77 ± 2.14 | 69.84 ± 5.94 | 75.67 ± 7.10 | 97.67 ± 6.06 | 95.18 ± 5.09 | 3.56 ± 0.44 | 11.18 ± 1.04 |
| **5** (20.0 kg, 30 N) | 9.98 ± 1.28 | 13.04 ± 1.82 | 22.14 ± 3.57 | 30.28 ± 3.76 | 31.61 ± 3.29 | 29.56 ± 2.68 | 3.61 ± 0.47 | 11.80 ± 1.33 |
| **6** (20.0 kg, 60 N) | 15.55 ± 1.94 | 15.79 ± 1.95 | 41.90 ± 4.86 | 52.72 ± 6.93 | 59.86 ± 6.74 | 57.00 ± 6.22 | 3.53 ± 0.44 | 11.81 ± 1.19 |
| **7** (20.0 kg, 90 N) | 17.07 ± 1.92 | 16.80 ± 1.93 | 57.08 ± 7.11 | 60.43 ± 6.21 | 83.61 ± 6.42 | 78.89 ± 5.67 | 3.58 ± 0.46 | 11.83 ± 1.24 |
| **8** (20.0 kg, 120 N) | 19.08 ± 2.16 | 17.30 ± 1.95 | 78.47 ± 8.45 | 73.54 ± 5.49 | 105.61 ± 5.61 | 101.49 ± 4.15 | 3.56 ± 0.46 | 11.76 ± 1.21 |
| **9** (25.0 kg, 30 N) | 12.76 ± 1.97 | 13.23 ± 1.76 | 24.73 ± 3.38 | 33.66 ± 3.72 | 38.61 ± 6.30 | 35.96 ± 5.94 | 3.53 ± 0.46 | 11.91 ± 1.26 |
| **10** (25.0 kg, 60 N) | 16.01 ± 2.26 | 16.50 ± 2.10 | 43.16 ± 6.23 | 53.66 ± 6.80 | 64.43 ± 6.02 | 61.41 ± 6.63 | 3.52 ± 0.46 | 12.06 ± 1.20 |
| **11** (25.0 kg, 90 N) | 17.96 ± 2.24 | 17.53 ± 2.25 | 61.67 ± 6.54 | 73.61 ± 9.31 | 86.80 ± 5.46 | 81.11 ± 4.89 | 3.69 ± 0.43 | 11.99 ± 1.15 |
| **12** (25.0 kg, 120 N) | 19.27 ± 2.33 | 17.48 ± 2.01 | 81.54 ± 9.63 | 82.13 ± 6.83 | 108.72 ± 5.21 | 104.16 ± 3.72 | 3.52 ± 0.46 | 12.12 ± 1.21 |

For each measured configuration, the mean of all subjects ± the standard error of measurement is shown.

* The configurations differ in load mass and tension to which the hip belt was adjusted, as shown in brackets.
